# Can Soil Covers Shield Farmland? Assessing Cadmium Migration Control from Coal Gangue Using a Multi-Compartment Approach

**DOI:** 10.3390/toxics13090717

**Published:** 2025-08-27

**Authors:** Hanbing Liu, Yao Feng, Chenning Deng, Zexin He, Huading Shi, Su Wang, Minghui Xie, Xu Liu

**Affiliations:** 1 School of Environmental Science and Engineering, Tianjin University, Tianjin 300072, China; 2 Technical Centre for Soil, Agriculture and Rural Ecology and Environment, Ministry of Ecology and Environment, Beijing 100012, China; 3 State Key Laboratory of Environmental Criteria and Risk Assessment, Chinese Research Academy of Environmental Sciences, Beijing 100012, China; 4 Nanjing Yangtze River Management Office, Nanjing 210009, China

**Keywords:** cadmium (Cd), coal mine, ecological risk, geoaccumulation index, Jiangxi Province

## Abstract

Potentially toxic element pollution caused by coal mining activities, especially the accumulation of cadmium, has become a major threat to the global environment and health. Long-term mining activities in China, a major coal consumer, caused a large accumulation of coal gangue. Gangue weathering and leaching release Cd, which threatens the ecological safety of the surrounding soil and water bodies. Although the government has implemented ecological restoration projects in the mining areas, there is still a lack of systematic evaluation of pollution control of downstream farmlands. For this study, remote sensing analyses of fractional vegetation coverage (FVC), geo-accumulation index (*I_geo_*), and potential ecological risk index (EI) data, as well as the pollution characteristics and ecological risks of Cd, were evaluated for a coal mining area in Jiangxi Province. Coal gangue, restoration cover soil, downstream farmland soil, irrigation water, and sediment samples were used in the analyses. After restoration, the Cd concentration in the mining cover soil (0.23 mg/kg) was significantly lower than that of the coal gangue (1.18 mg/kg), while the Cd concentration in the downstream farmland soil (0.44 mg/kg) was roughly an average of the two. The geo-accumulation index indicates that the farmland soil is mainly unpolluted (with an average *I_geo_* of −0.25). However, some points have reached the level of no pollution to moderate pollution. Coal gangue poses a relatively high ecological risk (with an average EI of 118), while cover soil and farmland soil pose low risks (with an average EI of 22.5 and 39.86, respectively). The restoration project significantly reduced the Cd input in the downstream farmlands. The study revealed the effective blocking of external soil cover on Cd migration, providing a key scientific basis for the optimization of ecological restoration strategy and risk prevention and control in similar mining areas worldwide.

## 1. Introduction

Cadmium bioaccumulates across ecosystems, contaminating air, water, soil, and crops. Its long-term persistence and non-degradability in soil amplify ecological and human health risks through chronic environmental exposure [1,2,3]. Rapid industrial development in coal mining areas and a lack of effective protective measures are the main causes of soil Cd pollution [4,5]. Coal consumption in China increased from 60 million tons in 1971 to 1.92 billion tons in 2015, and may reach its peak in 2050 [6,7]. The coal gangue produced during coal mining and beneficiation accounts for 10–15% of the total coal production, and is increasing at a rate of 200 million tons per year [8,9]. Cumulative coal gangue production will exceed 4.5 billion tons in China, occupying an area of more than 15,000 km^2^ [10]. During coal formation, Cd accumulates via (i) adsorption onto organic matter in anoxic peat swamps and (ii) co-precipitation with sulfide minerals under reducing conditions. Mining exposes coal and gangue to O_2_ and H_2_O, while the generated H^+^ dissolves Cd-bearing sulfides, releasing soluble Cd^2+^. Rainwater leaching then transports Cd into soils. This mechanism explains the elevated Cd in our gangue samples and its migration to downstream farmland [5]. Internationally, remediation strategies such as soil covers have demonstrated efficacy in isolating contaminants, yet their long-term effectiveness in acidic soils remains poorly quantified.

China started relatively late with the ecological restoration of quarries compared to developed countries, who began in the early 20th century. According to the “National Mineral Resources Plan (2008–2015)” of the Ministry of Land and Resources of China, the historical geological environmental problems of mines must be solved by raising funds through multiple channels to carry out restoration and governance [11]. By 2015, according to this plan, the restoration and governance rate of the mine’s geological environment should exceed 35%, and the reclamation rate of abandoned land from historical legacy mines should exceed 30%. This goal must be further enhanced by 2020 to achieve a recovery and governance rate of above 40%. The Ministry of Land and Resources further proposed the construction of several national green mines and green mining development demonstration zones in the National Mineral Resources Plan (2016–2020) [12]. China’s ‘green mine’ initiative drives ecological restoration in mining regions, directly supporting SDGs 15 (life on land) and 3 (good health). These efforts provide crucial support for reconciling environmental remediation with socioeconomic development. However, significant knowledge gaps persist regarding the effectiveness of restoration strategies—despite widespread adoption of policies encompassing engineering interventions (e.g., soil cover systems), chemical treatments, and microbial remediation. Some critical issues require further elaboration: How effectively does soil cover restoration mitigate cadmium mobility from coal gangue to downstream agricultural ecosystems, and what residual risks endure?

Extensive research has been done on ecological restoration technologies in mining areas, evaluation methods for potentially toxic element pollution, and pollution control strategies. Restoration technologies mainly include physical restoration, chemical restoration, biological restoration, or a combination of these technologies [13,14]. Potentially toxic element pollution evaluation often uses the geo-accumulation index and the potential ecological risk index [15,16,17]. These studies provide the theoretical basis for ecological restoration and technical support in mining areas. The findings offer scalable models for global mining sustainability, emphasizing source control to minimize residual contamination.

Even though ecological restoration has been achieved in some mining areas, the monitoring and evaluation of the restoration efforts are still insufficient. This study systematically investigates the environmental quality of irrigation water and several soils in the coal mining areas of Jiangxi Province, including coal gangue, new cover soil in the mining area, soil of downstream agricultural land, irrigation water, and sediment, especially for Cd. Methods such as fractional vegetation coverage, geo-accumulation index, and ecological risk index measurements were used to assess the effectiveness and potential ecological risks of the ecological restoration project in the Jiangxi Province mining area. This study was designed to quantitatively evaluate the effectiveness of soil cover restoration in mitigating cadmium (Cd) pollution across interconnected environmental compartments within Jiangxi’s coal mining region. The investigation pursued three specific objectives: (1) quantification of the multi-compartmental Cd distribution, involving determination of Cd concentrations and spatial patterns in coal gangue and restoration of cover soil, downstream farmland soils, irrigation water, and sediments to establish source–sink relationships; (2) assessment of ecological risk evolution, involving an evaluation of pre- and post-restoration ecological risks employing the geo-accumulation index (*I_geo_*) and potential ecological risk index (*E_r_*), supplemented by a temporal analysis of fractional vegetation coverage (FVC) trends (2000–2023) to assess ecosystem recovery dynamics; (3) identification of policy–implementation linkages, with the elucidation of persistent contamination pathways to derive actionable insights for optimizing remediation protocols under China’s ‘green mine’ initiative, with applicability to similar mining-affected regions globally.

## 2. Materials and Methods

### 2.1. Overview of the Study Area

Located in Jiangxi Province, Eastern China, the study area originally comprised undulating residual hill landforms characterized by weathered erosional features and well-developed gullies. Intensive human activities—notably mining and illicit opencast operations—have profoundly altered the natural topography. This mining area has been intensively exploited since the 1960s, with cumulative coal gangue production. Historical mining activities (1960s–2017) caused widespread land subsidence, deforestation, and acid drainage, directly contributing to Cd mobilization and exacerbating erosion and Cd transport to downstream farmland. Cd enrichment originates from Permian–Carboniferous coal seams rich in sulfides. Fractured karst aquifers further facilitate Cd migration into soils and groundwater. High rainfall (1565 mm/year) accelerates Cd leaching from gangue piles. Acidic soils increase Cd bioavailability, while seasonal floods disperse contaminants into the Ping River system. The perturbations include dismantled mountain structures, extensive vegetation clearance, and accumulated mining debris.

In 2021, ecological restoration for abandoned mines was carried out in the study area. Comprehensive prevention and control measures that were implemented in the mining area included waste residue landfill, terrain improvement, soil improvement, and crop planting efforts. The coal gangue was landfilled by external soil restoration, while the terrain of the mining area was transformed into multi-level wide platforms and gentle slopes. The remediation strategy employed a clean soil cover system, utilizing imported, uncontaminated external soil to cap coal gangue deposits. Application thicknesses were differentiated based on intended land use: 0.6 m for cropland, 0.4 m for dry farmland, and 0.3 m for forest land. This engineered barrier physically isolates cadmium (Cd)-rich gangue material from surface ecosystems, effectively mitigating leaching risks and preventing wind erosion of contaminated particulates. Land reconfiguration transformed slopes into multi-level terraced platforms to minimize runoff velocity and associated Cd migration. In paddy field areas, impermeable geotextile liners were installed as an additional hydraulic barrier to prevent Cd infiltration into underlying soil and groundwater. Complementary drainage infrastructure, including interception ditches and sedimentation ponds, was constructed to capture and contain Cd-laden surface runoff, enhancing the system’s overall contaminant containment capability. To achieve ecological restoration and land reuse in the mining area, planting soil and organic fertilizers were applied to improve the soil and ensure the growth of suitable crops. These efforts have improved both the geological environment of the mine and the land use value of the abandoned mining area.

### 2.2. Sample Collection and Testing

**Figure 1:**
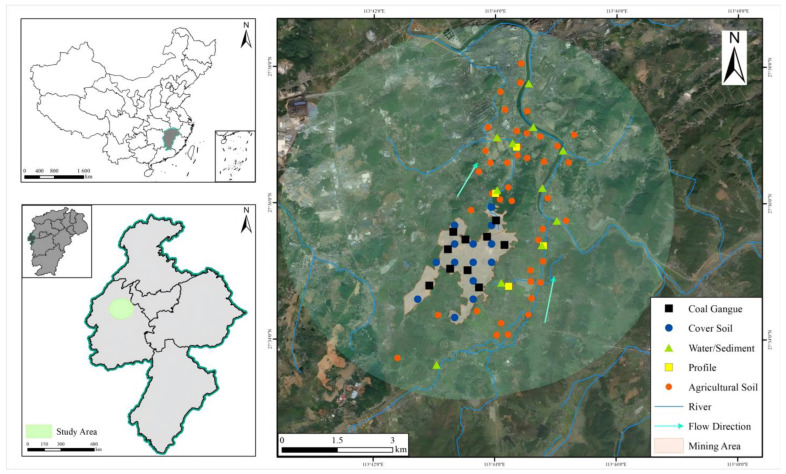
Schematic diagram of the sampling points in the study area.

Wooden shovels were used to collect surface soil samples. Before sampling, stainless steel and wooden shovels were placed into disposable plastic sealing bags to keep them clean. After the samples at each point were completed, the plastic sealing bags were replaced to ensure the cleanliness of the sampling tools and prevent cross-contamination between the different soil samples. To ensure an accurate assessment of the soil in the study area and the environmental effects of the restoration project on the soil of the surrounding farmland, the surface soil samples included both the cover soil of the mining area and the soil of the surrounding farmland (Figure 1). Twelve cover soil samples were collected within the mining area, 43 agricultural land soil samples were collected around the mining area, 11 samples were collected for both irrigation water and sediment, 10 coal gangue samples were collected, and 4 soil profiles were collected. Each soil sample comprised 5 subsamples composited within a 5 m radius. The total Cd concentration was tested for all these samples. Sampling objectives explicitly targeted quantifying cross-media contamination pathways. Coal gangue and cover soil analyses assessed source control effectiveness, while downstream farmland soils and irrigation water and sediment directly measured ecological transfer to agricultural systems. Soil profiles further revealed vertical heterogeneity of Cd enrichment, evidencing anthropogenic impacts beyond surface restoration (Table 1). All sites were mapped via GPS and integrated into project GIS.

Soil and sediment samples were air-dried, homogenized, and sieved (100-mesh) following HJ/T 166-2004 [18]. For the soil sample analysis, 0.3 g of homogenized sample was weighed into a PTFE crucible. Subsequently, 4 mL of hydrofluoric acid (HF), 5 mL of nitric acid (HNO_3_), and 2 mL of perchloric acid (HClO_4_) were added. The mixture was heated on a hot plate until dense white fumes appeared. Heating was continued until the solution clarified. The digestate was evaporated nearly to dryness. Finally, the residue was dissolved in 1% nitric acid (HNO_3_) and the solution was diluted to an appropriate volume for the analysis [19]. Water samples were filtered (0.45 μm), acidified to pH < 2, and pre-concentrated 10:1. Cadmium concentrations were determined using graphite furnace atomic absorption spectrometry (A3AFG-12, BGJC/YQ00301) per GB/T 17141-1997 (calibrated May 2022; LOD = 0.01 mg/kg), with operational parameters set to a 228.8 nm wavelength, 500 °C ashing, 1800 °C atomization, and argon carrier gas (99.999% purity) [20]. The instrument was calibrated before testing. The method accuracy was validated via spike recovery tests (84.7–96.2%) and certified reference material GBW07407a (89.1–96.2% recovery), while precision was confirmed through a parallel sample analysis (≤4.3% RSD). Detection limits were 0.0001 mg/L (water) and 0.01 mg/kg (soil/sediment). All the sampling personnel received relevant training and were familiar with the sampling process and precautions to ensure that sampling and testing were carried out strictly per technical specifications. Key field information such as the sampling points, time, and environmental conditions was recorded in detail during the sampling process. Calibrated instruments were used for testing, and the calibration certificate number and validity period were recorded. All test data were rechecked to ensure accuracy.

### 2.3. Assessment Methods for Potentially Toxic Element Pollution in the Soil

#### 2.3.1. Geo-Accumulation Index

The geo-accumulation index method can be used to estimate the natural changes in the distribution of potentially toxic elements and identify the impact of human activities [14]. The calculation is shown in Equation (1):

(1) $$I_{geo}=\log_{2}\left(\frac{C_{n}}{1.5B_{n}}\right)$$

C_n_ represents the measured concentration of the element in the sample, and B_n_ is the geochemical background value. The national agricultural land soil pollution risk screening value (0.3 mg/kg) replaced B_n_ in this study. The reason for choosing agricultural land standards is that these pollutants will eventually migrate to downstream farmland. The correction coefficient for changes in background data caused by geological background lithology and human activities was 1.5. The geo-accumulation index was divided into 7 categories: unpolluted (*I_geo_* ≤ 0), unpolluted to moderately polluted (0 < *I_geo_* ≤ 1), moderately polluted (1 < *I_geo_* ≤ 2), moderately polluted to polluted (2 < *I_geo_* ≤ 3), polluted (3 < *I_geo_* ≤ 4), very polluted to severely polluted (4 < *I_geo_* ≤ 5), and severely polluted (*I_geo_* > 5).

#### 2.3.2. Potential Ecological Risk Index

The potential ecological risk index that was used for pollution risk assessment is shown in Equation (2):

(2) $$E_{r}^{i}=T_{r}^{i}\times \frac{C^{i}}{C_{r}^{i}}$$

where *E^i^_r_* represents the potential ecological risk index of potentially toxic element *i* in the soil; *C_i_* represents the measured concentration of potentially toxic element *i* in the soil, in mg/kg; *C^i^_r_* represents the reference value of potentially toxic element *i*; *T^i^_r_*, which reflects the toxicity level of potentially toxic elements and the sensitivity of soil to these potentially toxic elements, is the toxicity response coefficient of potentially toxic elements (Cd = 30). Table 2 shows the classification standards used in this study [15].

The quality of Cd in coal gangue, cover soil, sediment, and farmland soil was evaluated using the ‘Soil Environmental Quality Risk Control Standard for Soil Contamination of Agricultural Land’ (GB 15618-2018) [21].

#### 2.3.3. Fractional Vegetation Coverage

The normalized difference vegetation index (NDVI) reflects the vegetation growth status [22]. The ratio of the near-infrared band to the red band image reflectance is usually used to extract vegetation information from multispectral images [23]. This can eliminate some radiation errors, and ranges from −1 to 1 [24]. The NDVI model was adopted to calculate the vegetation index of the study area in different years (Equation (3)).

(3) $$NDVI=\frac{\rho_{NIR}-\rho_{R}}{\rho_{NIR}+\rho_{R}}$$

where NDVI represents the normalized difference vegetation index, *NIR* is the reflectivity of the near-infrared band of the sensor, and *R* is the reflectivity of the thermal infrared band. Generally, the larger the NDVI in the green vegetation coverage area, the higher the surface fractional vegetation coverage.

The fractional vegetation cover (FVC) is a comprehensive quantitative indicator that can assess the regional ecological environment conditions of the vegetation coverage. Changes in the fractional vegetation coverage can also reflect the evolution of the mining area environment [25]. This paper extracted the FVC in coal mining areas using a dimidiate pixel model based on the NDVI. This is suitable for dynamic vegetation monitoring at a regional scale [26]. The principle of the dimidiate pixel model was used to obtain the FVC (see Equation (4)).

(4) $$FVC=\frac{NDVI-NDVI_{\min}}{NDVI_{\max}-NDVI_{\min}}$$

There is some variability in NDVI_min_ and NDVI_max_, which is affected by meteorological conditions, land feature distribution and type, and seasonal changes. It is necessary to delineate the confidence intervals of these terms based on the actual conditions of the study area before determining the upper and lower NDVI thresholds [27]. This paper used the NDVI_min_ and NDVI_max_ at 10% and 95% of the cumulative frequency of image elements.

Landsat 4-5 TM (2000–2011) and Landsat 8 OLI/TIRS (2012–2023) satellite imagery with 30 m spatial resolution was acquired from USGS EarthExplorer (cloud cover ≤ 5%). For NDVI calculations, band 3 (red, 0.63–0.69 μm) and band 4 (NIR, 0.76–0.90 μm) were used for Landsat 4-5, while band 4 (red) and band 5 (NIR) were used for Landsat 8. The images were downloaded and calculated from August to October each year, during the wet season with lush vegetation. The 2005, 2007, 2012, and 2014 years were excluded from the analyses due to the wide cloud coverage in these years. The FVC was classified into 5 types: extremely low coverage (0–0.2), low coverage (0.2–0.4), moderate coverage (0.4–0.6), high coverage (0.6–0.8), and extremely high coverage (0.8–1).

## 3. Results

### 3.1. Concentration of the Coal Gangue and Cover Soil in Mining Areas

#### 3.1.1. Coal Gangue Sample Analysis Results

Table 3 shows the characteristics of the Cd concentration in the coal gangue. The total Cd concentrations ranged between 0.50 and 1.50 mg/kg, with a median of 1.30 mg/kg. The average (1.18 mg/kg) was slightly lower than the median, indicating that there was no obvious maximum value of the Cd concentration in the coal gangue. There was also no bias in the data distribution. The Cd coefficient of variation in the coal gangue was 27.80%, showing medium variability. This suggests a relatively uniform Cd distribution in the coal gangue at different spatial positions. The Cd concentration in all the coal gangue samples exceeded the stipulated screening value and was lower than the control value. It exceeded the screening value by 1.25 to 4.00 times and had certain potential ecological risks.

#### 3.1.2. Cover Soil Sample Analysis Results

Table 4 shows the total Cd concentration in the soil of the mining area. The Cd concentrations ranged from 0.08 to 0.41 mg/kg. The average (0.23 mg/kg) was comparable to the median (0.21 mg/kg), indicating that the distribution of Cd concentrations in the external soil was robust, and there was no obvious extreme value point in space. The coefficient of variation of Cd in the soil of the mining area was moderate at 45.49%. The Cd concentration in the external cover soil was relatively homogeneous in its spatial distribution and reflected the basic characteristics of the region.

**Figure 2:**
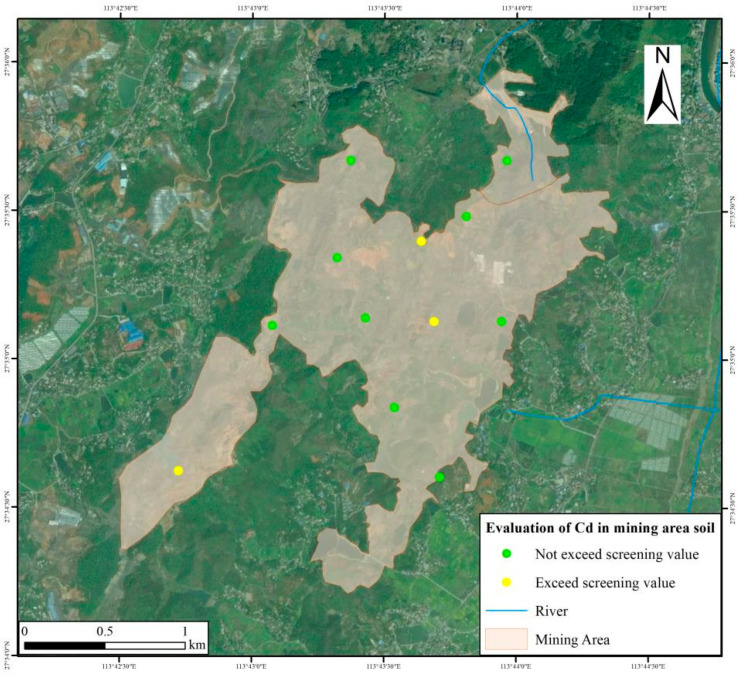
Comprehensive evaluation of soil environmental quality in the Dongguacao mining area.

There were 3 soil points in the mining area whose Cd concentrations exceeded the GB15618-2018 screening value by 1.03 to 1.37 times, but did not exceed the soil control value (Figure 2). Compared with coal gangue, the Cd concentration and pollution degree of the soil in the mining area have decreased significantly (Z = −3.964, *p* < 0.001), indicating effective restoration. The pH of the external soil in the mining area was acidic, ranging between 3.95 and 6.59 (Table 4). The release of potentially toxic elements is common for such pH ranges. Further evaluation is needed in the subsequent development and utilization process.

### 3.2. Cd Concentration of the Irrigation Water and Sediment

The concentration of Cd in soil is affected by both internal factors, such as the geological background, and man-made factors. Irrigation water, which is indispensable for agricultural production, also influences soil and affects elemental inputs in agricultural land. There are two irrigation ditches running through the mining area flow from southwest to northeast. The pH range of the 11 irrigation water samples collected along the ditches ranged from 6.7 to 7.2, and the Cd concentration was lower than the detection limit and the limit value stipulated in the “Standard for Irrigation Water Quality” (GB 5084-2021) [28] for all the samples. The undetectable Cd in irrigation water met all global standards, eliminating pathway risks.

The pH values of the sediment ranged from 4.02 to 8.19, with a moderately alkaline median of 7.99. The Cd concentrations ranged from 0.39 to 0.56 mg/kg, with an average of 0.48 mg/kg and a slightly higher median of 0.50 mg/kg (Table 5). This indicates that there was an extremely low Cd concentration in the sediment samples. Both the soil of the mining area and the cultivated land along the river affected the sediment source. Compared with the cover soil of the mining area, the sediment Cd concentration was relatively high. However, the sediment was in an alkaline environment with relatively stable physiochemical properties. The risk of mobilizing Cd is relatively low under such conditions. One sediment sample exceeded the GB 15618-2018 risk screening value, with an over-screening multiple of 1.7. No samples exceeded the control value.

### 3.3. Cd Pollution in the Surrounding Soil

**Figure 3:**
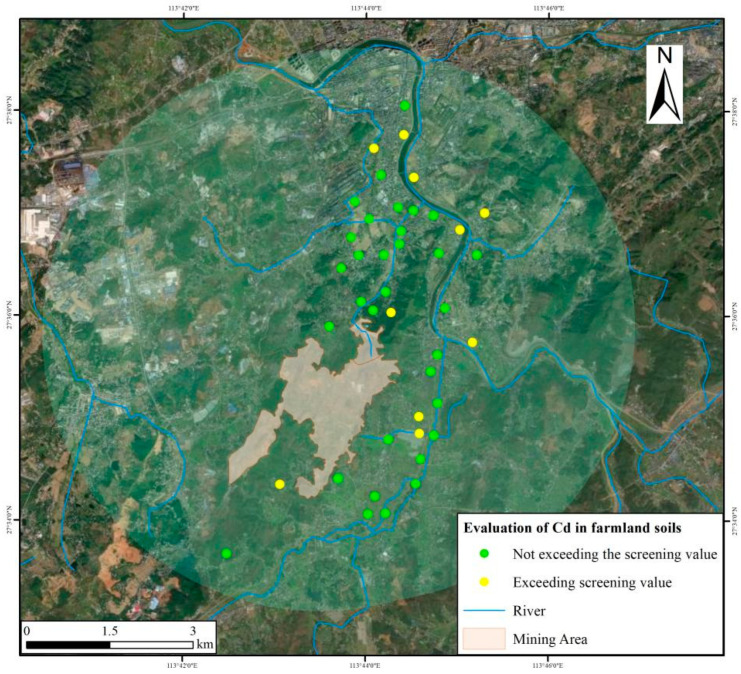
Cd evaluation of agricultural land soil around the mining area.

Table 6 shows the Cd concentrations in agricultural soil. The pH values of the surrounding farmland fluctuated greatly, ranging from 4.49 to 7.98, and the soil distribution ranged from acidic to alkaline. The Cd concentrations ranged between 0.10 and 0.57 mg/kg. The median (0.44 mg/kg) was comparable to the average (0.40 mg/kg), with a moderate coefficient of variation. The proportion of points exceeding the GB 15618-2018 Cd screening value was 22.7%, and the multiple range of the exceeding screening value was 1.13 to 1.83 (Figure 3). The proportion of the exceeding screening value decreased from the coal gangue to the bottom sediment and towards the downstream agricultural soils. Prominent acidification was observed in 10 soil samples that exceeded the screening values.

### 3.4. Vertical Variation of Cd in Farmland Soil Profiles

Vertical sampling revealed Cd enrichment in topsoil layers, directly evidencing recent anthropogenic inputs. Conversely, fluctuating concentrations in middle or deep strata reflect historical pollution phases, while stable low levels below 150 cm demarcate pre-mining geogenic baselines. Four soil profiles were collected beside the irrigation canals downstream of the mining area. Profiles TRPM01 and TRPM02 were close to the mining area, while profiles TRPM03 and TRPM04 were further away from the mining area.

**Figure 4:**
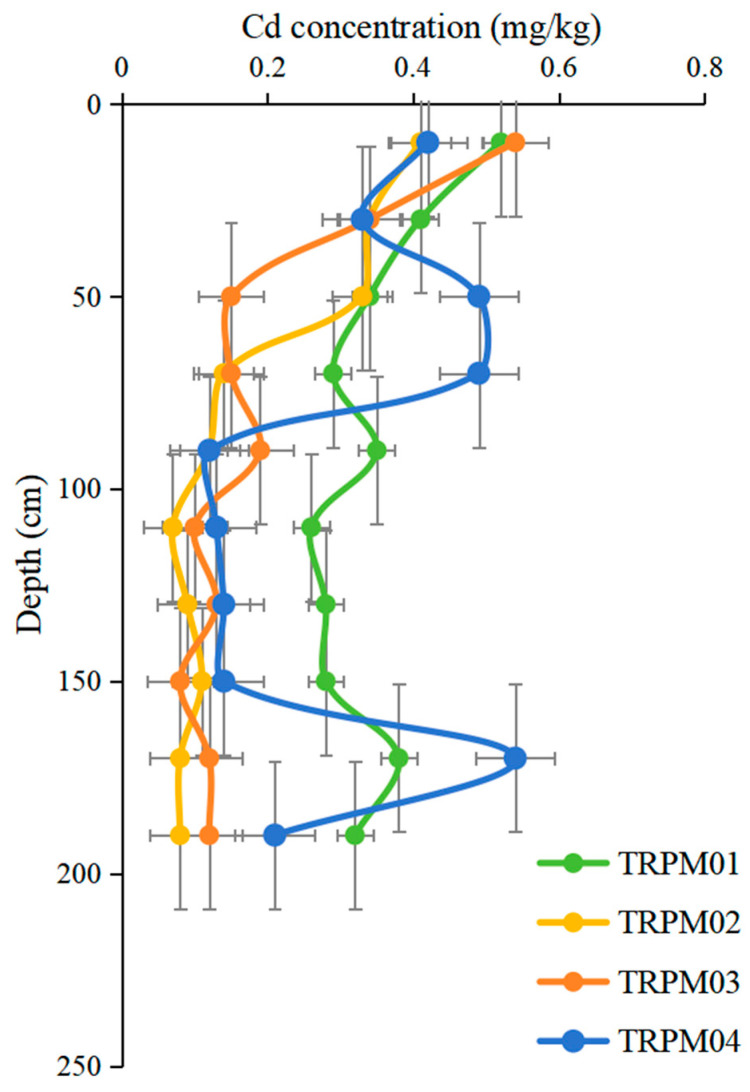
Vertical Cd variation of the profile.

For all four profiles, the Cd concentration in the topsoil was higher than in the bottom soil (Figure 4). The Cd concentration in the topsoil was 3.31 times that of the bottom soil, indicating obvious external input. There was a large fluctuation in Cd from the deep soil to the surface, indicating various processes such as the natural weathering of rocks and the input of pollution (coal gangue) during the mining process in the mining area. In the deep layer of the soil profiles, the Cd concentrations changed steadily (150–200 cm) at low levels, indicating that this process was mainly dominated by natural weathering. The middle and deep parts of the soil profiles showed varying degrees of fluctuation. This may indicate that both weathering and artificial disturbances, such as the leaching input of coal gangue from the upstream, affected the soil profiles at this depth. The highest values of all profile samples occurred at the surface layer. Despite the significant enrichment of Cd in the top layer of the soil profile, it is much lower than the soil pollution control value. Compared with the middle and deep parts of the soil profile, the surface soil Cd concentrations in one profile decreased. This means that after external soil restoration, the soils played a buffering role in the accumulation of Cd in the downstream soil. However, this also means that continuous monitoring is still needed to protect the quality of crops and human health in areas with relatively intense human activities such as cultivated land.

### 3.5. The Temporal Variation of Fractional Vegetation Coverage (FVC)

**Figure 5:**
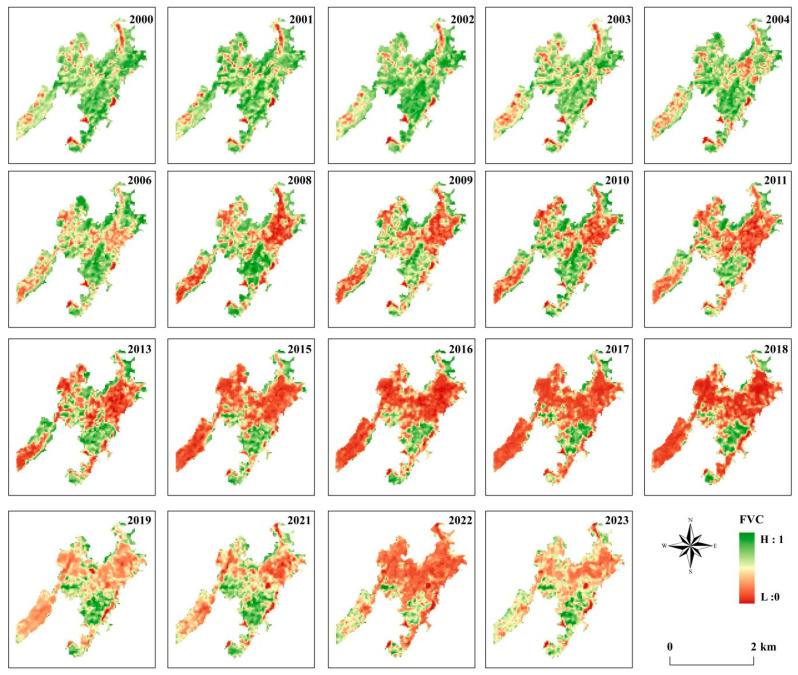
Classification of fractional vegetation coverage (FVC) in the mining area from 2000 to 2023.

ArcGIS was used to process the FVC of the study area from 2000 to 2023. Figure 5 shows the classification of the spatial variation characteristics of the FVC for the entire study area from 2000 to 2020. The FVC in the mining area fluctuated from 2000 to 2003 and decreased during the period from 2004 to 2016. The FVC reached its lowest value in 2017 (Figure 5).

## 4. Discussion

### 4.1. Heavy Metal Accumulation

Table 7 shows the calculation of the geo-accumulation index. The Cd concentration was the highest in the coal gangue, with an average geo-accumulation index of 1.32, showing moderate pollution. The Cd concentration was the lowest in the cover soil of the mining area, and the geo-accumulation index was negative, indicating that the soil environmental quality in the mining area was relatively high at present. The Cd concentration in the sediment was severely affected by the upstream coal gangue and the soil of the mining area, with a concentration between the coal gangue and the soil of the mining area. The average geo-accumulation index was 0.07, and this statistically significant decrease confirms restoration effectiveness in intercepting Cd transport. Some points in the agricultural land soil around the mining area were unpolluted to moderately polluted, accounting for 48.84%. These samples occurred in acidic, moderately acidic, and alkaline soils, indicating that this phenomenon was not influenced by external factors such as soil restoration. The pollution may have been due to the interference of leachate input from coal gangue in the early stage, and there was still a relatively high accumulated Cd concentration, presenting a certain risk.

The upstream coal gangue was exposed to the surface before the restoration project was implemented. A large amount of Cd in the reduced state would be released and migrate downstream with the water body due to weathering and leaching. When the hydrodynamic conditions were weak, Cd would be deposited in the sediment. Although the sediment is relatively stable under most conditions, secondary pollution can still occur when the hydrodynamic conditions are strong. Not only was the coal gangue removed from the source but the external soil covering also had lower Cd concentrations after the implementation of the restoration project, which greatly improved the downstream soil quality. Cd pollution around coal mines in northwestern Guizhou and western Chongqing, which are in the same climatic zone as Jiangxi, has reached moderate to high pollution levels [14,16]. This indicates that the Cd concentration in the farmland soil downstream of the mining area after restoration was of relatively good quality compared with the current status of the soil in other coal mining areas when considering the degree of weathering and leaching.

The geo-accumulation index (*I_geo_*) revealed distinct Cd pollution gradients; coal gangue exhibited moderate pollution (mean *I_geo_* = 1.32), while restored cover soils were unpolluted (*I_geo_* = −1.17), aligning with the effectiveness of soil-capping demonstrated in other coal mines. However, downstream farmland soils showed localized unpolluted-to-moderate contamination (*I_geo_* up to −0.25), contrasting sharply with heavily polluted mining areas in Guizhou (*I_geo_* > 0) [29].

### 4.2. Potential Ecological Risks

The toxicity coefficient of potentially toxic elements of the ecological risk index has an important reference value for the environmental assessment of potentially toxic elements in the study area. This study implemented a dual evaluation framework for Cd risks, combining regulatory soil screening values with contamination severity metrics. Table 8 shows the ecological risk assessment of different samples in the mining area. The ecological risk of Cd in the waste coal gangue was relatively high, while it was the lowest in the restored external soil. The combination of the two meant that the Cd in the soil and sediment of the surrounding farmland generally presented a low ecological risk. It is worth noting that 51.02% of the points in the surrounding farmland soil showed moderate ecological risk. This proportion was higher than the polluted proportion in the geo-accumulation index. This means that the unpolluted points are not safe when considering ecological effects. The toxic effects of Cd must also be fully considered when conducting crop production activities. An analysis of the geo-accumulation index and the ecological risk index suggests that the highly toxic Cd still poses a certain threat to the surrounding soil and water environment, even though the degree of pollution in the downstream environment of the mining area is relatively low.

Compared with the Cd risk levels in other coal mining areas (Table 8), various coal mining areas in Guizhou [30], Western Jiangxi [15], Jining, Shandong [31], and Jiang’an Sichuan [32] represent high-risk or even extremely high-risk points This study area had no high-risk area, indicating that the ecological restoration projects were very effective. However, some farmland soil and sediment still had moderate risk in the study area, indicating that although the external soil restoration has achieved remarkable results, it has caused relatively serious pollution during historical mining. Targeted governance is needed to achieve precise prevention and control going forward.

### 4.3. Ecological Restoration Analysis

After decades of high-intensity mining of the abundant coal resources in the study area, a series of negative impacts have occurred. These include occupying and destroying land resources, causing environmental pollution, inducing geological disasters, and causing ecological degradation. Generally speaking, ecological restoration in areas without human interference is slow, but in mining areas, it is an even more extensive and complex process. Remote sensing images from the period 2000 to 2003 (Table 9), the land area with relatively low FVC (0–0.4), increased over time from 35.91 hm^2^ in 2000 (accounting for 10.21% of the total land area) to 36.90 hm^2^ in 2003 (accounting for 10.54% of the total land area), indicating that the land had good coverage. From 2004 to 2018, the land with relatively low FVC soared to 70.35% but then decreased from 2019 to 2023 to 38.37%.

Similar patterns were observed in areas with high FVC (0.6–1), which increased from 206.64 hm^2^ in 2000 (accounting for 58.74% of the total land area) to 247.41 hm^2^ in 2002 (accounting for 70.32% of the total land area). From 2003 to 2018, the proportion of land with relatively high FVC dropped sharply to 17.11%, indicating that the FVC of the land deteriorated over time. However, from 2019 to 2023, it rose to 20.93%. In 2022, due to extreme drought weather conditions in Jiangxi, there were certain fluctuations.

As an important energy supply base in the region, the study area has undergone large-scale mining since 2006 and reached its peak in 2017. The mining activities have promoted economic development but also caused severe damage to the surrounding environment. Disorderly mining during this period extensively damaged the surrounding vegetation and disrupted the local ecological balance. The mined-out underground coal seams also triggered surface subsidence, causing land subsidence. The land subsidence caused cultivation difficulties and ecological degradation, and may trigger geological disasters. The increase in FVC within the mining area reflects an optimization of the vegetation community structure, increases in species diversity and vegetation coverage, the fixation of soil by vegetation roots, reductions in water and soil losses, and improvements of the soil fertility and structure [33]. Improved vegetation coverage provides a habitat and food source for wild animals, restores biodiversity, and forms a more complete ecosystem.

Fractional vegetation coverage (FVC) trends (2000–2023) correlated with pre-restoration (2006–2021) Cd contamination levels, where a relatively high decline to 3.81% (2021) coincided with peak ecological risk. Post-restoration (2021–2023), FVC recovery to 20.93% paralleled reduced Cd mobility in cover soil (0.23 vs. 1.18 mg/kg in gangue) and decreased downstream farmland *E_r_* (22.5 vs. 118), demonstrating that vegetation restoration directly suppressed contaminant dispersion. The integration of geo-accumulation index (*I_geo_*), ecological risk (*E_r_*), and fractional vegetation coverage (FVC) data revealed that soil-capping reduced Cd mobility short-term (*I_geo_* cover soil = −1.17 vs. gangue = 1.32; *E_r_* drop from 118 to 22.5), yet residual moderate risk persists in 51% of farmlands (*E_r_* = 40–80) due to accumulation through history.

### 4.4. Research Implications

Coal mining activities have long caused serious pollution and ecological degradation. It has become one of the most urgent environmental problems worldwide [4,5,34]. The main restoration strategies for coal mine pollution are highly similar for different geographical and climatic conditions of mining areas in different regions [12]. Of these, external soil restoration technology is an effective means of governance. Covering contaminated soil with uncontaminated soil reduces direct exposure to pollution sources, thereby curbing further environmental deterioration [35].

Ecological restoration projects in the study area showed clear ecological and environmental benefits. The comprehensive evaluation of the previous geo-accumulation index method and the potential ecological risk index method were used to identify the source–pathway–sink migration law of potentially toxic element pollutants and the spatial differentiation characteristics of ecological risks. Results show that ecological restoration can address pollution, with a focus on demonstrating its environmental response mechanism and the effect of collaborative governance. The recovery path of the ecosystem’s self-purification capacity under artificial intervention has been revealed by considering the environmental monitoring data of the mining area together with the FVC. This confirmed that the systematic restoration plan based on the source–pathway–sink theory was effective in addressing the pollution, and provided a scientific basis for the sustainable improvement of the ecological environment in the mining area and its surrounding areas.

While our risk assessment quantified soil Cd reductions, the study lacks empirical data on Cd uptake in crops and native vegetation. This gap prevents full characterization of trophic transfer risks in restored ecosystems. Meanwhile, three critical limitations warrant acknowledgment: (1) the acidic pH of cover soils may exacerbate future Cd mobility through leaching; (2) temporal FVC variability reveals fluctuating vegetation recovery, indicating restoration benefits are climate-dependent and vulnerable to extreme droughts; (3) sample size constraints limit statistical power for spatial extrapolation, although vertical soil profiles and multi-media sampling partially mitigate this through process-based validation. Future monitoring will prioritize: (1) Cd accumulation in staple crops, (2) bioavailability assays via DGT techniques, and (3) isotopic tracing to differentiate legacy vs. new contamination distinction.

## 5. Conclusions

This study systematically evaluated potentially toxic element pollution and ecological risks in a coal mine in Jiangxi Province. The occurrence characteristics of cadmium in different environmental media presented significant differences. The average concentration of Cd was the highest in the coal gangue and the lowest in the restored external soil. The concentration in the downstream soil and sediment was between the two. The Cd concentration of some acidic soils exceeded the national standard. The geo-accumulation index showed that the farmland soil was generally unpolluted. However, some points ranged from unpolluted to moderately polluted due to the influence of historical coal gangue accumulation. The Cd with a high toxicity coefficient in coal gangue posed a relatively high ecological risk. The average ecological risks of the soil in the mining area, farmland soil, and sediment were all low, confirming that the external soil and restoration project has achieved remarkable results. These results advance restoration science by quantifying soil cover systems as critical barriers against toxic metal flux.

This study systematically evaluated the effectiveness of soil cover restoration in controlling cadmium contamination within coal mining areas, demonstrating its significant attenuation of Cd migration risks to downstream farmland ecosystems. These findings directly advance the core objectives of China’s ‘green mine’ initiative—achieving ecological rehabilitation and pollution control through optimized engineering strategies—while providing empirical evidence for advancing the United Nations Sustainable Development Goals. While affirming the soil cover system’s efficacy in reducing Cd migration, this study proposes three actionable measures for policymakers and operators under China’s ‘green mine’ initiative: (1) mandate pH buffering for acidic cover soils to prevent long-term Cd mobilization; (2) implement continuous topsoil monitoring in farmlands, prioritizing sites with historical enrichment; (3) adopt isotope tracing to distinguish legacy vs. new contamination sources.

## Figures and Tables

**Table 1:** Sample depth and collection protocol.

Sample Type	Spatial Strategy	Selection Criteria
Coal Gangue	Stratified random sampling across gangue piles, 500 g	Represent weathering variability
Cover Soil	Grid (200 × 200 m) over restored areas, 0–20 cm, 500 g	Uniform coverage of soil cover depth/texture
Farmland Soil	Grid (500 × 500 m) over irrigation flow paths, 0–20 cm, 500 g	Downstream Cd migration gradient
Irrigation Water	Paired with farmland sites, 1 L	Direct exposure to crops
Sediment	Same as water samples, 500 g	Water-Sediment contamination
Soil Profiles	Strategic downstream positions near canals, 500 g	Sampling at 20-cm intervals

**Table 2:** Classification standards for potential ecological risks.

Potential Ecological Risk Index	Risk Level
*E_r_* < 40	Very low ecological risk
40 ≤ *E_r_* < 80	Low ecological risk
80 ≤ *E_r_* < 160	Moderate ecological risk
160 ≤ *E_r_* < 320	High ecological risk
*E_r_* ≥ 320	Extreme ecological risk

**Table 3:** Characteristics of Cd in coal cangue (mg/kg).

Monitoring Indicators	Concentration						Coefficient of Variation/%
	Minimum	25% Percentile	Median	75% Percentile	Maximum	Mean	
pH	2.89	5.39	6.52	6.89	7.76	6.08	22.99
Cd	0.50	1.05	1.30	1.40	1.50	1.18	27.80

**Table 4:** Concentrations of Cd in the new cover soil of the mining area (mg/kg).

Monitoring Indicators	Concentration						Coefficient of Variation/%
	Minimum	25% Percentile	Median	75% Percentile	Maximum	Mean	
pH	3.95	4.15	4.40	4.59	6.59	4.53	14.67
Cd	0.08	0.15	0.21	0.30	0.41	0.23	45.49

**Table 5:** Concentrations of Cd in sediment (mg/kg).

Monitoring Indicators	Concentration						Coefficient of Variation/%
	Minimum	25% Percentile	Median	75% Percentile	Maximum	Mean	
pH	4.02	7.43	7.99	8.06	8.19	7.47	15.67
Cd	0.39	0.43	0.50	0.52	0.56	0.48	11.92

**Table 6:** Concentrations of Cd in agricultural soil (mg/kg).

Monitoring Indicators	Concentration						Coefficient of Variation/%
	Minimum	25% Percentile	Median	75% Percentile	Maximum	Mean	
pH	4.49	6.02	6.83	7.53	7.98	6.73	13.55
Cd	0.10	0.34	0.44	0.48	0.57	0.40	29.17

**Table 7:** The geo-accumulation index analysis of different samples.

		Coal Gangue	Soil in the Mining Area	Sediment	Soil of Agricultural Land
*I_geo_*	Minimum	0.15	−2.49	−0.21	−2.17
	Average	1.32	−1.17	0.07	−0.25
	Maximum	1.74	−0.13	0.32	0.34
Average pollution degree		Moderately polluted	Unpolluted	Unpolluted to Moderately polluted	Unpolluted

**Table 8:** The analysis of ecological risk index values of different samples.

		Coal Gangue	Soil in the Mining Area	Sediment	Surrounding Soil
*EI*	Minimum	50.00	8.00	39.00	10.00
	Average	118.00	22.50	47.73	39.86
	Maximum	150.00	41.00	56.00	57.00
Average ecological risk level		Medium ecological risk	Low ecological risk	Moderate ecological risk	Low ecological risk
Comparison of Cd risk levels in other mining areas/%		Guizhou	A coal mine in Jiangxi Province	Jining City	Jiang ‘an County
Extremely low ecological risk		37.78	0	6.49	15.89
Low ecological risk		6.66	0	75.33	47.66
Moderate ecological risk		17.77	17.24	15.58	33.65
High ecological risk		35.55	37.93	2.60	2.80
Extremely high ecological risk		2.22	44.83	0	0

**Table 9:** Areas of vegetation coverage categories in different parts of the mining area for different periods (unit: hm^2^).

Year	0–0.2		0.2–0.4		0–0.4		0.4–0.6		0.6–0.8		0.8–1		0.6–1	
	Area	Proportion	Area	Proportion	Area	Proportion	Area	Proportion	Area	Proportion	Area	Proportion	Area	Proportion
2000	6.57	1.87%	29.34	8.34%	35.91	10.21%	109.26	31.06%	191.34	54.39%	15.30	4.35%	206.64	58.74%
2001	3.87	1.10%	26.55	7.55%	30.42	8.65%	94.59	26.89%	199.80	56.79%	27.00	7.67%	226.80	64.47%
2002	6.21	1.77%	31.77	9.03%	37.98	10.80%	66.42	18.88%	184.50	52.44%	62.91	17.88%	247.41	70.32%
2003	4.14	1.18%	32.76	9.36%	36.90	10.54%	117.27	33.50%	171.81	49.09%	24.03	6.87%	195.84	55.95%
2004	5.31	1.51%	54.81	15.58%	60.12	17.09%	104.76	29.78%	163.71	46.53%	23.22	6.60%	186.93	53.13%
2006	3.06	0.87%	61.11	17.37%	64.17	18.24%	123.57	35.12%	141.30	40.16%	22.77	6.47%	164.07	46.64%
2008	28.35	8.06%	92.52	26.30%	120.87	34.36%	82.35	23.41%	100.71	28.63%	47.88	13.61%	148.59	42.24%
2009	1.62	0.46%	106.20	30.19%	107.82	30.65%	98.82	28.09%	117.63	33.44%	27.54	7.83%	145.17	41.26%
2010	54.00	15.35%	83.97	23.87%	137.97	39.22%	69.57	19.77%	96.66	27.48%	47.61	13.53%	144.27	41.01%
2011	62.19	17.68%	113.04	32.13%	175.23	49.81%	70.11	19.93%	79.29	22.54%	27.18	7.73%	106.47	30.26%
2013	35.19	10.00%	120.96	34.38%	156.15	44.38%	64.80	18.42%	96.48	27.42%	34.38	9.77%	130.86	37.20%
2015	109.71	31.18%	104.22	29.62%	213.93	60.81%	53.91	15.32%	64.35	18.29%	19.62	5.58%	83.97	23.87%
2016	75.96	21.59%	148.41	42.18%	224.37	63.78%	62.64	17.81%	50.22	14.27%	14.58	4.14%	64.80	18.42%
2017	34.02	9.67%	198.45	56.41%	232.47	66.08%	55.80	15.86%	40.50	11.51%	23.04	6.55%	63.54	18.06%
2018	158.40	45.02%	89.10	25.33%	247.50	70.35%	44.10	12.54%	39.87	11.33%	20.34	5.78%	60.21	17.11%
2019	1.35	0.38%	39.60	11.26%	40.95	11.64%	195.48	55.56%	86.85	24.69%	28.53	8.11%	115.38	32.80%
2021	29.34	8.34%	107.28	30.49%	136.62	38.83%	116.91	33.23%	82.08	23.33%	16.20	4.60%	98.28	27.94%
2022	54.45	15.48%	200.16	56.89%	254.61	72.37%	83.79	23.82%	12.06	3.43%	1.35	0.38%	13.41	3.81%
2023	9.54	2.71%	125.46	35.66%	135.00	38.37%	143.19	40.70%	62.28	17.70%	11.34	3.22%	73.62	20.93%

## Data Availability

Data are contained within the article.
